# Measurement Invariance and Intergenerational Parallelism of General Self-Efficacy in Adolescent and Parent Dyads

**DOI:** 10.3389/fpsyg.2020.01251

**Published:** 2020-06-04

**Authors:** Hui Lei, Yanyun Yuan, Zhihang Wang, Jianshi Chen, Zhihua Li

**Affiliations:** ^1^College of Education, Hunan Agricultural University, Changsha, China; ^2^College of Teacher Education, Ningbo University, Ningbo, China

**Keywords:** GSE, general self-efficacy, measurement invariance, intergenerational parallelism, socioeconomic status

## Abstract

General self-efficacy refers to global beliefs about one’s capabilities across a variety of tasks or conditions. It is regarded as an important, relatively stable, motivational trait, and is associated with positive outcomes in a wide range of domains. The general self-efficacy scale (GSE) is the most commonly used measure to evaluate general self-efficacy among adults and youths. This study aimed to examine the measurement invariance of the GSE across age groups among adolescent and parent dyads, and to investigate the intergenerational parallelism of general self-efficacy and the moderating roles of parents’ gender and family socioeconomic status (SES). Participants were 807 adolescent/parent dyads. Multi-group confirmatory factor analysis revealed that full metric and scalar measurement invariance held. Regression analysis showed that parents’ self-efficacy significantly predicted their children’s self-efficacy (β = 0.232, *p* < 0.001). Multiple regression analysis indicated family SES played a moderating role (β = 0.066, *p* < 0.001), although parents’ gender did not (β = −0.053, *p* = 0.288). The results demonstrated the GSE’s measurement invariance across age, and further supported use of the GSE among adults and adolescents. Moreover, our findings provided evidence for the presence of this kind of intergenerational parallelism and the moderating role of family SES.

## Introduction

Self-efficacy is defined as a personal perceived capability to accomplish specific tasks to achieve the desired results. Perceived self-efficacy is concerned not with the number of skills you have, but with what you believe you can do with what you have under a variety of circumstances (Bandura, 1997). It has been considered to substantially affect how people feel, think, and behave (Bandura, 1997, 1999, 2000). Specifically, persons with high self-efficacy have high self-esteem, and low levels of depression, helplessness, and anxiety. They have a strong sense of competence that facilitates cognitive processes and performance in various tasks. Additionally, they tend to choose challenging tasks, and invest more effort in and persist longer in pursuing their goals than those with low self-efficacy (Bandura, 1997; Scholz et al., 2002). Self-efficacy is suggested to be a significant predictor of various positive outcomes, such as performance accomplishments and emotional well-being, and has increasingly received attention in psychology (Lazi and Jovanovi, 2018).

To evaluate the concept effectively, researchers have commonly used measures of two kinds of self-efficacy: state-like or task-specific and trait-like general self-efficacy. The former version is based on the view that people have a set of relatively stable self-beliefs in different areas or specific situations of functioning (Bandura, 1997, 2012). The latter concerns a broader concept of self-efficacy that refers to global confidence in one’s capacity across a variety of tasks or conditions (Schwarzer, 1992). General self-efficacy is regarded as an important, relatively stable, motivational trait, that is more likely to be associated with positive outcomes in a wide range of domains (Tipton and Worthington, 1984; Chen et al., 2001).

The general self-efficacy scale (GSE) developed by Schwarzer and Jerusalem (1995) is the most commonly used measure to evaluate general self-efficacy. It has been translated into over 30 languages and used in different cultural contexts. Thus, its psychometric properties have been extensively evaluated. For instance, Scholz et al. (2002) examined the structural validity of the GSE in 25 countries and confirmed its unidimensional structure across cultures. Some researchers provided evidence about measurement invariance across groups and time. For example, Teo and Kam (2014) tested the GSE’s measurement invariance across two different cultures. Other studies investigated measurement invariance across gender (Leung and Leung, 2011; Lazi and Jovanovi, 2018) and over time (Grevenstein and Bluemke, 2017; Lazi and Jovanovi, 2018). However, there is a lack of evidence for the GSE’s measurement invariance across age, even though the GSE scale has been frequently used in different age groups, including adults (Zhang and Schwarzer, 1995; Scholz et al., 2002; Luszczynska et al., 2005; Schwarzer and Jerusalem, 2010; Lazi and Jovanovi, 2018) and adolescents (Meilstrup et al., 2016; Chang et al., 2018; Lönnfjord and Hagquist, 2018; Sun et al., 2018).

According to Bandura’s Social Cognitive Theory, self-efficacy beliefs are developed from four major sources, including personal accomplishment or mastery, vicarious experience, symbolic experience, and emotional arousal (Bandura, 1977, 1986). Vicarious experience refers to observing a “model person” who is similar to oneself, and who successfully deals with the difficulty confronted. The model’s successes can enhance self-efficacy beliefs by social comparison processes. For children and adolescents, their parents are vital and effective social models who contribute to fostering their self-efficacy beliefs. Symbolic experience means that individuals are persuaded by others’ verbal encouragement to believe that they are able to succeed. Parents are also important sources of symbolic experience. Thus, parents with high general self-efficacy could raise their children with high general self-efficacy through providing a positive “model person” who confronts life stress with high perceived self-efficacy, and provides more verbal encouragement during their children’s upbringing. Intergenerational parallelism refers to comparable feelings, cognitions, or behaviors across generations (Kaplan et al., 2006). To our knowledge, there is little empirical evidence to support the existence of intergenerational parallelism of general self-efficacy. Lin (2003) found that parents’ self-efficacy during adolescence had a strong influence on the development of their children’s self-efficacy, indicating an intergenerational parallelism of self-efficacy. However, Moskowitz (1992) failed to find intergenerational parallelism of self-efficacy across three generations.

Therefore, the present research had two principal aims: (1) to examine the measurement invariance across age among adolescent and parent dyads; (2) to investigate the intergenerational parallelism of general self-efficacy by examining the association between parents’ and adolescents’ general self-efficacy. Moreover, we considered two moderating variables (family socioeconomic status and parents ‘gender) in the second purpose. This was based on the following considerations. Some previous studies have found gender differences in GSE scores. For instance, Scholz et al. (2002) reported that women have higher GSE scores than men. In addition, family socioeconomic status is an important environmental factor that affects parenting behaviors, and in turn, influences adolescents’ self-efficacy (Whitbeck et al., 2006).

## Materials and Methods

### Participants

Participants comprised 807 adolescent/parent dyads recruited from 36 high and primary schools in five cities (Changsha, Loudi, Hengyang, Chengzhou, and Shaoyang) in Hunan province, China. A letter of introduction about the research was sent to each schools’ principal, and then consent was obtained from the schools. The investigators met with parents of students who were interested in participating in this research, and presented the study protocol. Informed consent, including their own and the children’s permission to participate, was obtained from the parents. This study underwent an ethical review and was approved by the Academic Committee of the College of Education of Hunan Agricultural University. The parent sample consisted of 431 women (53.4%) and 376 men (46.6%). Their age ranged from 26 to 59 (*M* = 40.38, *SD* = 5.58). The adolescent subjects consisted of 482 girls (59.7%) and 325 boys (40.3%). The youths’ age ranged from 7 to 17 (*M* = 11.63, *SD* = 2.51).

### Measures

#### The General Self-Efficacy Scale

The General Self-Efficacy Scale (GSE) is a 10-item self-report measure developed by Schwarzer and Jerusalem (1995). Responses range from 1 (not at all true) to 4 (exactly true). We used a Chinese version of the scale (Zhang and Schwarzer, 1995). The internal consistency for this version is α = 0.87. In the current study, internal consistency for the GSE was 0.864 in the adolescent sample and 0.865 in the parent sample.

#### Family Socioeconomic Status

Family socioeconomic status (SES) was assessed using the family monthly income reported by parents and the parents’ education level. The family monthly income ranged from 1 (“less than 1000 *yuan* per month”) to 8 (“more than 10,000 *yuan* per month”). The father’s and mother’s highest level of education was divided into six categories: illiteracy (1), elementary school (2), junior high school (3), senior high school or technical secondary school (4), undergraduate education (5), postgraduate education (6). The parents’ education level and family monthly per month were normalized. The family SES score equaled the sum of both normalized scores. Specifically, one subject’s family monthly income minus the mean of family monthly income, and then the difference divided by its standard deviation was the *Z* score. The *Z* score of parent’s educational level was got by the same formula. The sum of two *Z* scores was the subject’s family SES score.

### Data Analysis

Exploratory factor analysis (EFA) was conducted on parent and adolescent samples using SPSS version 21 to explore the dimensionality of the GSE scale. Principal components analysis was used to extract the factors. Two decision rules (Scree plot and parallel analysis) were used to identify the number of factors to retain. A confirmatory factor analysis (CFA) and a Multi-Group CFA were conducted with Mplus 6.12 (Muthén, 1998-2011). The parameter estimates were obtained using maximum likelihood parameter estimates with standard errors and a mean-adjusted chi-square test statistic. Several fit indices were used to evaluate the model: the Comparative Fit Index (CFI), the Tucker–Lewis Index (TLI), the root-mean-square error of approximation (RMSEA), and the standardized root mean squared residual (SRMR). The model is considered to be acceptable if RMSEA ≤ 0.08, SRMR ≤ 0.08, TLI ≥ 0.90, and CFI ≥ 0.09 (Hu and Bentler, 1999).

Measurement invariance of the GSE across age was tested by means of a multi-group CFA, which consisted of a hierarchical set of four steps (Samuel et al., 2015). In the first step, configural invariance was tested by setting factor loadings, intercepts of variables, and residual variances as free parameters. Second, metric invariance refers to constraints of equivalent factor loadings and release of intercepts and residual variances. Third, we constrained the factor loadings and intercepts to be equal across groups to test scalar invariance. Finally, the strict or residual invariance was tested by constraining factor loadings, intercepts of variables, and residual variances that were all set to be equal across groups.

The changes in CFI (ΔCFI) and RMSEA (ΔRMSEA) values between alternative models were used to evaluate the fit of nested models, due to the limitations of Δχ^2^, which is highly sensitive to sample size (Schermelleh-Engel et al., 2003): a ΔCFI ≤ 0.010 and ΔRMSEA ≤ 0.15 were considered evidence of invariance (Chen, 2007).

A hierarchical multiple regression analysis was conducted to identify whether parents’ general self-efficacy contributed to their sons’ or daughters’ general self-efficacy and whether family SES or parents’ gender served as a moderator. The regression analysis was performed in two steps: family SES and general self-efficacy were entered into the regression (step 1), the interaction terms (the cross product of the two variables) were added into the regression model (step 2). The parents’ general self-efficacy and family SES were standardized before being added into the regression model. The moderating effect of parents’ gender was tested by the same procedure.

## Results

### Descriptive Statistics

The characteristics of the two groups are presented in Table 1. No significant difference in gender ratio was found between the adolescent sample and parent sample (*χ^2^* = 0.217, *p* = 0.222). The GSE item means did not differ significantly between the adolescent and parent samples (*t* = 1.66, *p* = 0.097). We tested the difference in item means of GSE between girls (*M* = 2.64, *SD* = 0.55) and boys (*M* = 2.68, *SD* = 0.54) in the adolescent sample and between mothers (*M* = 2.61, *SD* = 0.51) and fathers (*M* = 2.62, *SD* = 0.51) in the parent sample, and no difference was observed (Adolescent: *t* = 0.95, *p* = 0.344; Parent: *t* = 0.14, *p* = 0.885).

**TABLE 1:** Characteristics of the Adolescent parent Dyads (*N* = 807).

	Adolescent	Parent	*t/χ^2^*	*p*
Age *M*(*SD*) range	11.63 (2.51)	40.38 (5.85)		
	7–17	26–59		
**Gender *n*(%)**				
Male	325 (40.3)	376 (46.6)	0.217	0.222
Female	482 (59.7)	431 (53.4)		
SES *M*(*SD*)		3.10 (1.67)		
**Marital status *n*(%)**				
Married		668 (82.8)		
Separate		22 (2.7)		
Divorced		76 (9.4)		
Widowed		41 (5.1)		
**GSE**				
*M*(*SD*)	2.65 (0.54)	2.61 (0.51)	1.66	0.097

*SES = Socioeconomic status; GSE = general self-efficacy.*

### Building the Baseline Model

For the adolescent samples, the KMO value (0.904) and Bartlett’s test of sphericity (*p* < 0.001) confirmed the factorability of the data set. Both the scree plot and parallel analysis supported the one-factor structure. All of items loaded strongly on one factor (loadings ranged from 0.629 to 0.715). One-factor structure was also confirmed in the patient samples (loadings ranged from 0.557 to 0.725). The factorial loadings were presented in the Table 2. We also tested the one-factor structure using CFA The fit indices are presented in Table 3. The original one-factor model of the GSE showed a good fit based on CFI and SRMR, but was not supported by TLI and RMSEA. The inspection of residual moments indicated that there were strong correlated residuals between item 4 (I am confident that I could deal efficiently with unexpected events) and 5 (Thanks to my resourcefulness, I can handle unforeseen situations), which is one pair of similarly worded items. So the residuals of these two items were correlated to improve model fit. The following indices demonstrated global goodness of fit for the refined one-factor model: the CFI values were > 0.90; the TLI values were > 0.90; the RMSEA values were < 0.08; and the SRMR values were < 0.08. These fit indices indicate a good fit of the single-factor model for each sample. Moreover, the internal consistency for GSE was α = 0.864 in the adolescent sample and α = 0.865 in the parent sample. Corrected item-total correlation coefficients ranged from 0.523 to 0.619 in the adolescent group and from 0.471 to 0.634 in the parent group. Thus, the one-factor model was set as the baseline model.

**TABLE 2:** Exploratory factor analysis for the GES scale with one-factor solution.

Items	Factorial loading	
	Adolescents group	Parents group
(1) I can always manage to solve difficult problems if I try hard enough	0.715	0.725
(2) If someone opposes me, I can find the means and ways to get what I want	0.705	0.724
(3) It is easy for me to stick to my aims and accomplish my goals	0.689	0.723
(4) I am confident that I could deal efficiently with unexpected events	0.688	0.716
(5) Thanks to my resourcefulness, I know how to handle unforeseen situations	0.675	0.716
(6) I can remain calm when facing difficulties because I can rely on my coping abilities	0.664	0.698
(7) I can solve most problems if I invest the necessary effort	0.657	0.684
(8) When I am confronted with a problem, I can usually find several solutions	0.657	0.599
(9) If I am in trouble, I can usually think of a solution	0.648	0.586
(10) I can usually handle whatever comes my way	0.629	0.557
*Total variance explained (percent)*	45.30	45.67

**TABLE 3:** Goodness of fit indices for the baseline model.

	*SBχ^2^*	*df*	RMSEA	SRMR	CFI	TLI
*Original one-factor model*						
Adolescents	226.740	35	0.084	0.051	0.911	0.882
Parents	228.135	35	0.083	0.053	0.907	0.881
*Refined one-factor Model*						
Adolescents	188.336	34	0.076	0.047	0.928	0.902
Parents	191.158	34	0.076	0.051	0.925	0.900

*SBχ^2^ = Satorra-Bentler robust χ^2^; RMSEA = robust root mean square error of approximation; SRMR = standardized root mean squared residual; CFI = comparative fit index; TLI = Tucker–Lewis index.*

### Testing for Measurement Invariance of the GSE

The refined one-factor model was used as a baseline model. The results of the measurement invariance tests across age are shown in Table 4. Both the RMSEA statistic and CFI index indicated that the configural model (Model 1) yielded a good fit to the data (RMSEA = 0.080, CFI = 0.918). Next, we assessed the metric invariance model (Model 2), and did not find a significant difference between the configural and metric models (ΔRMSEA = 0.003; ΔCFI = 0.007). Then we tested the scalar invariance (Model 3). The results supported it because no significant difference was found between the metric and scalar models (ΔRMSEA = 0.001; ΔCFI = 0.008). Finally, the strict invariance (Model 4) was examined. It was not supported by a significant difference between the scalar and strict models (ΔCFI = 0.015), although the ΔRMSEA = −0.002. We released item 9, which had the largest difference in residual variance between the two groups. Additionally, the partial strict invariance (Model 5) yielded a good fit (ΔRMSEA = 0.000; ΔCFI = 0.008).

**TABLE 4:** Measurement invariance (MI) test of the GSE scale in adolescent and adult sample.

Model	*χ^2^*	*df*	RMSEA	CFI	TLI	Model comparison	ΔRMSEA	ΔCFI	ΔTLI
(1) Configural invariance	416.081	70	0.080	0.918	0.891				
(2) Metric invariance	445.516	79	0.077	0.913	0.898	vs. M1	0.003	0.005	−0.007
(3) Scalar invariance	489.996	88	0.076	0.905	0.900	vs. M2	0.001	0.008	−0.002
(4) Strict invariance	563.321	98	0.078	0.890	0.897	vs. M3	−0.002	0.015	0.003
(5) Partial M4	532.711	97	0.076	0.897	0.902	vs. M4	0.000	0.008	−0.002

*RMSEA = robust root mean square error of approximation; CFI = comparative fit index; TLI = Tucker–Lewis index. Model 1 refers to the configural invariant factor model; Model 2 refers to the metric invariant factor model; Model 3 refers to a scalar invariant factor model; Model 4 refers to the strict invariant factor model; Model 5 refers to a partial strict invariant factor model.*

### Predictors of Adolescent Self-Efficacy

The mean scores of the GSE items in the adolescent group were set as the dependent variable, and the mean scores in the parent group as the independent variable. Regression analysis showed that parents’ self-efficacy significantly predicted their children’s self-efficacy (β = 0.232, *p* < 0.001; *R*^2^ = 0.054, *F*(1, 805) = 45.96, *p* < 0.001).

We first evaluated the moderating effect of family SES on the relation between parents’ self-efficacy and their offspring’s self-efficacy using hierarchical multiple regression analysis. As shown in Table 5, parents’ self-efficacy (β = 0.228, *p* < 0.001) and family SES (β = 0.066, *p* < 0.001) were both positively related to adolescents’ self-efficacy. The interaction effect between parents’ self-efficacy and SES on adolescents’ self-efficacy was significantly positive (β = 0.0.07, *p* < 0.05), and explained an additional 0.5% of variance in adolescents’ self-efficacy (Δ*R*^2^ = 0.005; Δ*F*(1, 803) = 4.137, *p* < 0.05). This indicates that family SES played a moderating role in the relation between parents’ self-efficacy and adolescents’ self-efficacy.

**TABLE 5:** Hierarchical multiple regression analysis testing SES as moderating the relationship between GSE for parents and GSE for adolescents.

	GSE_A							
Predictor	*B*	*B*_*se*_	*β*	*R*	*R*^2^	Δ*R*^2^	Δ*F*	*p*
Step 1				0.244	0.060	0.060	25.534	<0.001
GSE_P	0.223	0.034	0.223***					
SES	0.076	0.034	0.076*					
Step 2				0.254	0.065	0.005	4.137	0.042
GSE_P * SES	0.075	0.037	0.070*					

SES = Socioeconomic status; GSE = general self-efficacy; GSE_P = GSE for parents; GSE_A = GSE for adolescents. *p < 0.05; ***p < 0.001.

Next, the moderating effect of parents’ gender was tested. Results showed that the main effect of parents’ self-efficacy for children’s self-efficacy still was significant (β = 0.272, *p* < 0.001), however, the main effect of gender (β = 0.074, *p* = 0.062) and the interaction effect between parents’ self-efficacy and gender was not significant (β = −0.053, *p* = 0.288; Δ*F*(1, 803) = 1.128, *p* = 0.288), indicating a non-significant moderating role of parents’ gender.

Lastly, the moderating effect of parents’ age and adolescents’ age separately was evaluated. We did not find a significant interaction effect of parents’ age (β = −0.003, *p* = 0.288; Δ*F*(1, 803) = 0.008, *p* = 0.928) and adolescents’ age (β = −0.003, *p* = 0.288; Δ*F*(1, 803) = 0.008, *p* = 0.928).

## Discussion

The purpose of the current study was twofold: (1) to examine measurement invariance of the GSE scale across age; (2) to investigate the intergenerational parallelism of general self-efficacy by examining whether the parents’ general self-efficacy predicted adolescents’ general self-efficacy. Moreover, we evaluate the moderating effects of gender and SES.

As far as we know, this is the first research to investigate the GSE’s measurement invariance across age. The GSE scale displayed adequate item discrimination and internal consistency reliability and EFA for both samples supported the underlying unidimensional model for this scale. The findings of CFA showed that a single-factor structure for the GSE achieved good fit to the data, indicating that the GSE scale is a unidimensional measure of generalized beliefs about one’s capability of coping with a wide range of demanding or novel situations. This is consistent with previous studies (Zhang and Schwarzer, 1995; Scholz et al., 2002; Luszczynska et al., 2005; Schwarzer and Jerusalem, 2010; Lazi and Jovanovi, 2018). In addition, the presence of configural invariance across age suggested that the GSE scale is a unidimensional measure for both adults and adolescents.

In this study, our findings provided support for metric and scalar invariance across age, which indicates that factor loadings and intercepts were invariant across two different age groups. The partial strict invariance was supported by releasing the residual variance of one item to be variant across groups. The existence of full metric and scalar invariance suggests that the observed mean differences in the GSE scores can be fully explained by the mean differences in the latent factor named general self-efficacy. Evidence of partial strict invariance indicates the validity of investigating the strength of the associations between the latent variable of the GSE and other constructs across age. The GSE scale demonstrated adequate reliability and validity in adults (Zhang and Schwarzer, 1995; Scholz et al., 2002; Luszczynska et al., 2005; Schwarzer and Jerusalem, 2010; Lazi and Jovanovi, 2018) and adolescents (Chang et al., 2018; Lönnfjord and Hagquist, 2018; Sun et al., 2018), with a lack of evidence for measurement invariance across age. Our findings provided evidence regarding this psychometric property.

We examined the intergenerational parallelism of general self-efficacy and found that parents’ GSE were relatively weak predictors of adolescents’ GSE. This finding is partially in accordance with results reported by Lin (2003). Lin (2003) evaluated self-efficacy using two observed indicators (locus of control and perceived control over one’s environment), and found that first generation self-efficacy in adolescence significantly predicted second generation adolescent self-efficacy. Our findings provide further support for theoretical expectations in terms of general self-efficacy parallelism across generations. Adolescents acquire much self-efficacy information from their families and home environments (Schunk and Miller, 2002). Parents with high general self-efficacy could provide a positive “model person” and more verbal encouragement to raise their children’s self-efficacy beliefs. Moreover, parents who are most successful in promoting positive competence perceptions are able to modify their demands and expectations according to the changing needs, abilities, and dispositions of children as they develop (Eccles et al., 1998). This facilitates them having more successful experiences and then developing stronger self-efficacy beliefs. In addition, parental educational attainment and expectation was found to mediate the intergenerational parallelism of self-efficacy (Lin, 2003).

Furthermore, we found that family SES positively moderated the intergenerational parallelism in general self-efficacy. Families with high SES are more likely to provide educational resources to their children, which consequently promote their academic and career development. Additionally, parents of higher SES families are more likely to inspire their children, which results in them having stronger self-efficacy beliefs in their future academic endeavors and subsequent careers (Lin, 2003). It should be noted that these results do not mean that all children from poor families develop low self-efficacy. Family SES is a descriptive rather than an explanatory variable in this case. In addition, in line with a previous study (Lin, 2003), our study showed that parents’ gender did not moderate the intergenerational parallelism in general self-efficacy. Parents’ gender also does not moderate the intergenerational transmission of other aspects of the self, such as self-concept clarity (Crocetti et al., 2016). Given the age range in both groups is wide, we also evaluated the moderating effect of parents’ and adolescents’ age. Both variables did not play moderating role, which suggested that the age range could not affect the relation between parents’ self-efficacy and their offspring’s self-efficacy.

The current study has several limitations. First, our research sample is Chinese, so the issue of whether the measurement invariance of the GSE scale across age holds in other cultural contexts needs to be tested. Second, our cross-sectional research design makes it difficult to interpret causal effects, although our data for both generations were provided separately by their own samples, and our results supported the existence of an intergenerational parallelism of general self-efficacy. However, it is possible that development of general self-efficacy among two generations is influenced by common experiences from similar family SES or social circumstances. In the future, a longitudinal research design should be used to uncover whether intergenerational parallelism of general self-efficacy is an intergenerational transmission phenomenon. Third, our work did not address the question of how the first generation’s self-efficacy influences self-efficacy in the second generation. Additionally, further studies on mediating factors are recommended.

In sum, our work demonstrated for the first time the measurement invariance of the GSE scale across age, and further supported the use of the GSE among adults and adolescents, which is a meaningful contribution to the measurement of general self-efficacy. We also focused on the intergenerational parallelism in general self-efficacy. The results of our research provide evidence for the presence of this kind of intergenerational parallelism and the moderating role of family SES. These findings lend support for the notion that parents’ general self-efficacy plays an important role in the development of their children’s general self-efficacy.

## Data Availability Statement

The raw data supporting the conclusions of this article will be made available by the authors, without undue reservation, to any qualified researcher.

## Ethics Statement

The study was reviewed and approved by the Academic Committee of the College of Education of Hunan Agricultural University. Written informed consent to participate in this study was provided by the participants’ legal guardian/next of kin.

## Author Contributions

ZL was mainly responsible for the overall conception, design of this study. HL wrote the manuscript and carried out the statistical analysis. YY, ZW, and JC were responsible for the questionnaire survey and sorting out.

## Conflict of Interest

The authors declare that the research was conducted in the absence of any commercial or financial relationships that could be construed as a potential conflict of interest.
